# Evolutionary conservation of Zinc-Activated Channel (ZAC) functionality in mammals: a range of mammalian ZACs assemble into cell surface-expressed functional receptors

**DOI:** 10.3389/fmolb.2023.1265429

**Published:** 2023-09-04

**Authors:** Anders A. Jensen

**Affiliations:** Department of Drug Design and Pharmacology, Faculty of Health and Medical Sciences, University of Copenhagen, Copenhagen, Denmark

**Keywords:** pentameric ligand-gated ion channel (pLGIC), Zinc-Activated Channel (ZAC), two-electrode voltage-clamp electrophysiology (TEVC), mammalian, evolutionary conservation, zinc, proton

## Abstract

In contrast to the other pentameric ligand-gated ion channels in the Cys-loop receptor superfamily, the *ZACN* gene encoding for the Zinc-Activated Channel (ZAC) is exclusively found in the mammalian genome. Human ZAC assembles into homomeric cation-selective channels gated by Zn^2+^, Cu^2+^ and H^+^, but the function of the receptor in human physiology is presently poorly understood. In this study, the degree of evolutionary conservation of a functional ZAC in mammals was probed by investigating the abilities of a selection of ZACs from 10 other mammalian species than human to be expressed at the protein level and assemble into cell surface-expressed functional receptors in mammalian cells and in *Xenopus* oocytes. In an enzyme-linked immunosorbent assay, transient transfections of tsA201 cells with cDNAs of hemagglutinin (HA)-epitope-tagged versions of these 10 ZACs resulted in robust total expression and cell surface expression levels of all proteins. Moreover, injection of cRNAs for 6 of these ZACs in oocytes resulted in the formation of functional receptors in two-electrode voltage-clamp recordings. The ZACs exhibited robust current amplitudes in response to Zn^2+^ (10 mM) and H^+^ (pH 4.0), and the concentration-response relationships displayed by Zn^2+^ at these channels were largely comparable to that at human ZAC. In conclusion, the findings suggest that the functionality of ZAC at the molecular level may be conserved throughout mammalian species, and that the channel thus may govern physiological functions in mammals, including humans.

## 1 Introduction

The four subfamilies of classical pentameric ligand-gated ion channels (pLGICs) in the Cys-loop receptor superfamily mediate the fast transmission of acetylcholine (ACh), serotonin (5-hydroxytryptamine, 5-HT), γ-aminobutyric acid (GABA) and glycine (Alexander et al., 2021). The nicotinic ACh, 5-HT_3_, GABA_A_ and glycine receptors (nAChRs, 5-HT_3_Rs, GABA_A_Rs, and GlyRs, respectively) are abundantly expressed in the CNS and in several peripheral tissues, where they mediate a plethora of processes (Taly et al., 2009; Chua and Chebib, 2017; Lynch et al., 2017; Gibbs and Chakrapani, 2021). Because of their direct involvement in pathologies or the potential they offer for pharmacological intervention in these, the classical pLGICs have been pursued as therapeutic targets for a range of indications, and several marketed drugs against insomnia, epilepsy, anxiety and nausea/emesis act through the receptors (Ghasemi and Hadipour-Niktarash, 2015; Lynch et al., 2017; Fakhfouri et al., 2019; Belelli et al., 2020; Juza et al., 2020; Terry and Callahan, 2020; Gibbs and Chakrapani, 2021).

In contrast to this extensive research into the classical pLGICs, the physiological functions governed by the Zinc-Activated Channel (ZAC), the only member of the fifth subfamily of mammalian pLGICs, are still unknown. While the ZAC protein exhibits very low homology (<20% amino acid sequence identity) to the other superfamily members, ZAC comprises most of the structural hallmarks of a pLGIC subunit (Davies et al., 2003). Moreover, expression of human ZAC in mammalian cells and *Xenopus* oocytes leads to formation of homomeric cation-selective channels gated by zinc (Zn^2+^), cupper (Cu^2+^) and protons (H^+^) (Davies et al., 2003; Trattnig et al., 2016; Madjroh et al., 2021a). The channel properties displayed by the recombinant human ZAC differ substantially from of most classical pLGICs, as the receptor exhibits substantial spontaneous activity and very slow activation and desensitization kinetics (Davies et al., 2003; Trattnig et al., 2016; Madjroh et al., 2021a).

The human ZAC is encoded by the *ZACN* gene at chromosome 17q23 which is derived from the same ancestral gene as the nAChRs and 5-HT_3_Rs, and orthologous *ZACN* genes are found in most mammalian genomes published to date. Interestingly, however, *ZACN* genes are absent from the rat and mouse genomes, which has hampered explorations of the physiological functions and therapeutic potential of ZAC since its discovery (Davies et al., 2003). ZAC is expressed at the transcript level in human brain, pancreas, placenta, thyroid, prostate, trachea and stomach tissue (Davies et al., 2003; Houtani et al., 2005), and immunostaining data has suggested ZAC protein expression in human thymus, lymph organs and regulatory T cells (Chang, 2019). Moreover, the overall high conservation of the *ZACN* gene in the mammalian genomes seems to be indicative of a function for ZAC in mammalian physiology. However, this would obviously require that ZAC functionality at the molecular level be retained throughout the mammalian species. In the present study, it was investigated whether ZACs from a range of other mammalian species than human are expressed at the protein level and assemble into cell surface-expressed functional receptor complexes in mammalian cells and in *Xenopus* oocytes.

## 2 Materials and methods

### 2.1 Materials

ZnCl_2_ (0.1 M solution buffered at pH 7.4), poly-*D*-lysine (PDL), and chemicals for buffers were purchased from Sigma-Aldrich (St. Louis, MO), and culture medium, serum, antibiotics and trypsin for cell culture was obtained from Invitrogen (Paisley, UK). Defolliculated stage V-VI oocytes harvested from female *Xenopus laevis* frogs were obtained from an in-house facility.

### 2.2 Molecular biology

**FIGURE 1:**
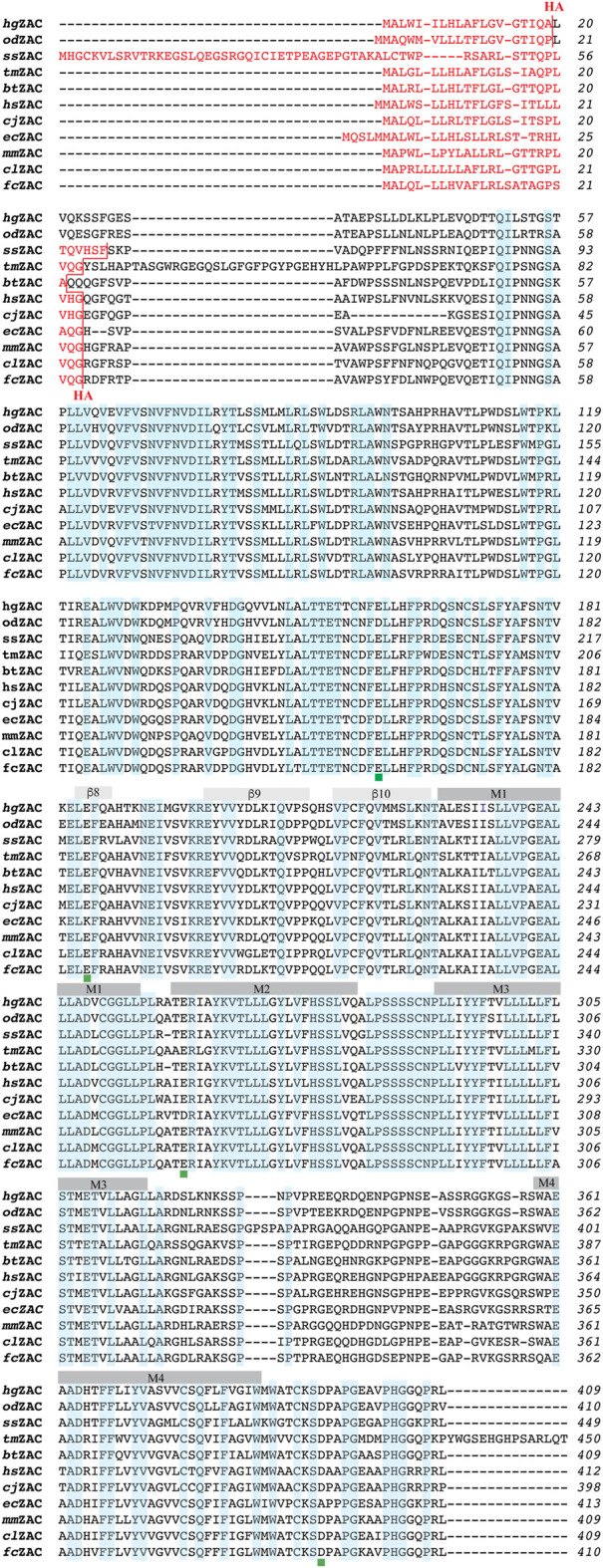
Alignment of the amino acid sequences of 11 ZACs from different mammalian species: *hs*ZAC, *cj*ZAC, *cl*ZAC, *fc*ZAC, *ec*ZAC, *bt*ZAC, *ss*ZAC, *mm*ZAC, *od*ZAC, *hg*ZAC, and *tm*ZAC. The sequence alignment was performed using the web-based Clustal Omega program. The signal peptides in the ZAC proteins are given in red font, and the HA-epitope insertion points in the proteins are indicated (in red). The ten β-sheets (β1-10) in the extracellular domains and the four α-helices (M1-4) in the transmembrane domains of the proteins are indicated. The amino acid residues that are completely conserved in all 11 ZACs are highlighted in light blue. The positions of the four candidate protonation residues conserved in the six H^+^-gated ZACs (*hs*ZAC*, cj*ZAC, *cl*ZAC, *bt*ZAC, *hg*ZAC and *tm*ZAC) but not in *ec*ZAC are indicated with green squares below the alignments.

The construction of hemagglutinin (HA) epitope-tagged human ZAC (hereafter termed *hs*ZAC) in pUNIV and of the 5-HT3B-pCIneo and HA-5-HT3B-pCIneo plasmids has been reported previously (Krzywkowski et al., 2007; Krzywkowski et al., 2008; Madjroh et al., 2021a). The cDNAs for HA-tagged versions of ZAC subunits from 10 other mammalian species inserted between the *Nhe*I and *EcoR*I restriction sites in the multicloning site of pUNIV were purchased from Genscript (Piscataway, NJ). These HA-tagged ZACs that were used for all experiments in this work will hereafter be termed *cj*ZAC, *cl*ZAC, *fc*ZAC, *ec*ZAC, *bt*ZAC, *ss*ZAC, *mm*ZAC, *od*ZAC, *hg*ZAC, and *tm*ZAC (Table 1). In the HA-tagged ZAC cDNAs, a 27-nucleotide sequence (5′-TAC​CCA​TAC​GAT​GTT​CCA​GAT​TAC​GCT-3′) encoding for the HA epitope (Tyr-Pro-Tyr-Asp-Val-Pro-Asp-Tyr-Ala) was inserted between the nucleotide sequences for the predicted signal peptide and the predicted mature ZAC protein, analogously to the position of the HA-epitope in *hs*ZAC. The signal peptide predictions were made using the Signal P 5.0 software (Almagro Armenteros et al., 2019). Except for the incorporation of optimized Kozak sequences in all 11 HA-ZAC cDNAs, none of the ZAC cDNAs were optimized for expression in heterologous expression systems. Details about the mammalian species for these ZAC subunits and the insertion points of the HA-tag sequence in the cDNAs are given in Table 1, and a protein sequence alignment of the 11 ZAC proteins are given in Figure 1.

**TABLE 1:** The *hs*ZAC and the 10 ZACs from other mammalian species studied in this study. The name of the HA-tagged ZAC subunit, the species and the mammalian order it belongs to, the GenBank accession number for the cDNA, and the insertion points of the HA epitope in the respective subunit (at the protein level) are given.

Receptor	Species	Mammalian Order	Accession No.	Insertion of HA epitope
*hs*ZAC	*Homo sapiens* (human)	Primates (*Primata*)	NM_180990	-V-H-G-∣∣-Q-G-F-
*cj*ZAC	*Callithrix jacchus* (common marmoset)	Primates (*Primata*)	XM_008997774	-V-H-G-∣∣-E-G-F-
*cl*ZAC	*Canis lupus* familiaris (domestic dog)	Carnivores (*Carnivora*)	NM_001010955	-V-Q-G-∣∣-R-G-F-
*fc*ZAC	*Felis catus* (domestic cat)	Carnivores (*Carnivora*)	NM_011289384	-V-Q-G-∣∣-R-D-F-
*ec*ZAC	*Equus ferus caballus* (domestic horse)	Odd-toed ungulates (*Perissodactyla*)	NM_014737539	-A-Q-G-∣∣-H-S-V-
*bt*ZAC	*Bos taurus* (domestic cow)	Even-toed ungulates (*Artiodactyla*)	NM_001191252	-P-L-A-∣∣-Q-Q-Q-
*ss*ZAC	*Sus scrofa* (domestic pig)	Even-toed ungulates (*Artiodactyla*)	XM_021066603	-H-S-F-∣∣-S-K-P-
*mm*ZAC	*Molossus molossus* (velvety free-tailed bat)	Bats (*Chiroptera*)	XM_036269489.1	-V-Q-G-∣∣-H-G-F-
*od*ZAC	*Octodon degus* (common degu)	Rodents (*Rodentia*)	XM_004630211	-I-Q-P-∣∣-L-V-Q-
*hg*ZAC	*Heterocephalus glaber* (naked mole-rat)	Rodents (*Rodentia*)	XM_004861068	-I-Q-A-∣∣-L-V-Q-
*tm*ZAC	*Trichechus manatus* (West Indian manatee)	Sea cows (*Sirenia*)	XM_004374386	-V-Q-G-∣∣-Y-S-L-

### 2.3 Enzyme-linked immunosorbent assay (ELISA)

The total and cell surface expression levels of *hs*ZAC, *cj*ZAC, *cl*ZAC, *fc*ZAC, *ec*ZAC, *bt*ZAC, *ss*ZAC, *mm*ZAC, *od*ZAC, *hg*ZAC and *tm*ZAC transiently expressed in tsA201 cells were quantified using an ELISA. The tsA201 cells were cultured in GlutaMAX-I Dulbecco’s Modified Eagle’s Medium supplemented with 10% fetal bovine serum, penicillin (100 U/mL) and streptomycin (100 μg/mL) in a humidified atmosphere of 5% CO_2_ at 37°C. Exponentially growing cells were split out into 6 cm tissue culture dishes (1 × 10^6^ cells/dish) and transfected 20–24 h later with 4.0 μg cDNA for the respective HA-ZAC subunits using PolyFect (Qiagen, West Sussex, UK) according to the manufacturer’s protocol. Parallel transfections of cells with 6 cm tissue culture dishes with “empty” pUNIV vector or with HA-5-HT3A-pCIneo (both 4.0 μg cDNA per 6 cm dish) were used as “mock” controls (assessing non-specific binding of the antibody) and as reference for normalization, respectively. Cells transfected with HA-5-HT3B-pCIneo/pCIneo (2.0 μg/2.0 μg per 6 cm dish) and with HA-5-HT3B-pCIneo/5-HT3A-pCIneo (2.0 μg/2.0 μg per 6 cm dish) were used as controls for cell surface expression relative to total cell expression of a HA-tagged subunit.

16–18 h after the transfection, the cells were plated into PDL-coated 48-well plates (1 × 10^5^ cells/well). The ELISA experiment was initiated 24–25 h after the plating of the cells, and it was performed essentially as previously described (Krzywkowski et al., 2007; Madjroh et al., 2021a). The cells were washed two times with ice-cold wash buffer (phosphate-buffered saline supplemented with 1 mM CaCl_2_) and incubated in an ice-cold 4% paraformaldehyde solution for 12 min on ice, after which the following steps were performed at RT. The cells were washed three times with wash buffer, incubated for 30 min in blocking solution (3% dry milk in 50 mM Tris-HCl, 1 mM CaCl_2_, pH 7.5), and incubated with rat monoclonal anti-HA-peroxidase conjugated antibody (clone 3F10, Sigma-Aldrich, diluted 1:1,000 in blocking solution) for 1 h. Total expression levels of the HA-tagged proteins in the cells were determined in parallel wells by adding 0.1% Triton X-100 in the blocking solution used during the blocking and antibody-incubation steps. Cells were then washed four times with wash buffer, and expression of the HA-tagged proteins was quantified using 3,3′,5,5′-tetramethylbenzidine liquid substrate system (Sigma Aldrich) and H_2_SO_4_. The absorbance of the supernatants was determined at 450 nm. All experiments were performed in triplicate, and data are based on a total of three independent experiments.

### 2.4 *Xenopus laevis* oocytes and two-electrode voltage clamp (TEVC) recordings

The functional properties of six HA-ZACs (*cj*ZAC, *cl*ZAC, *ec*ZAC, *bt*ZAC, *hg*ZAC and *tm*ZAC) expressed in *Xenopus* oocytes were studied and compared to those of *hs*ZAC in TEVC recordings. The cDNAs for the ZACs (all in the pUNIV vector) were linearized with *Not*I and subsequently transcribed and capped using the mMessage mMachine T7 RNA transcription kit (Ambion, Waltham, MA). Stocks of 0.05 μg/μL were prepared for almost all cRNAs, and volumes of 18.4 and 36.8 nL (i.e., cRNA quantities of 0.92 and 1.84 ng) were injected into the oocytes. Oocytes were incubated in a sterile modified Barth’s solution [88 mM NaCl, 1 mM KCl, 15 mM HEPES (pH 7.5), 2.4 mM NaHCO_3_, 0.41 mM CaCl_2_, 0.82 mM MgSO_4_, 0.3 mM Ca(NO_3_)_2_, 100 U/mL penicillin and 100 μg/mL streptomycin] at 16°C–18°C, and the oocytes were used 2–3 days after the injection.

On the day of experiment, all compound dilutions were prepared in a saline solution [115 mM NaCl, 2.5 mM KCl, 10 mM MOPS (pH 7.5), 1.8 mM CaCl_2_, 0.1 mM MgCl_2_] and pH was adjusted to 7.5 (if needed). Thus, with exception of those applications testing H^+^ as ZAC agonist, all TEVC recordings were performed at pH 7.5. Oocytes were placed in a recording chamber continuously perfused with this saline solution, and the agonist were applied in the perfusate. Both voltage and current electrodes were agar-plugged with 3 M KCl with a resistance of 0.2–2.0 MΩ. Oocytes were voltage-clamped at −50 mV by a Gene Clamp 500B amplifier and current signals were digitized by a Digidata 1322A (both from Axon Instruments, Union City, CA). Currents were recorded using pCLAMP 10 (Molecular Devices, Sunnyvale, CA). The recordings were performed at room temperature.

In the TEVC recordings, the agonists Zn^2+^ or H^+^ were applied in the bath until the peak current decayed to a steady state or for a time period up to 30 s. Due to slow activation kinetics not all concentrations of the agonists produced a peak current within 30 s at all of the ZACs, analogously to what previously also has been observed for wild-type (untagged) human ZAC in oocyte recordings (Madjroh et al., 2021a), To be able to determine a complete agonist concentration-response relationship on an oocyte, the maximal current evoked by each agonist concentration within a 30 s-application period was extracted as the data. Although this represents an approximation, the true pharmacological properties of the ZAC ligands are likely to be well represented by this data analysis. At the beginning or end of all recordings determining concentration-response relationships at ZAC, two consecutive applications of 10 mM Zn^2+^ giving rise to a maximal agonist-induced current (I_max_) were applied on the oocyte, and it was verified that these consecutive applications elicited responses of comparable current amplitudes (±20%).

### 2.5 Data analysis

Analysis of the data from the TEVC recordings were performed using Clampfit software version 10.5 (Molecular Devices, Crawley, UK) and GraphPad Prism version 8.0 (GraphPad Software, La Jolla, CA). Concentration-response curves for Zn^2+^ were fitted in Prism by nonlinear regression using the equation for sigmoidal dose-response with variable slope. Each data point represents the mean ± S.E.M. value of recordings performed on at least five oocytes in total from at least two different batches. For the ELISA data, the average absorbance in the wells with tsA201 cells transfected with pCIneo (4.0 μg, “mock” control) were subtracted from the absorbance measured in all other wells, which subsequently were normalized to the cell surface expression measured in the wells with cells transfected with HA-5-HT3A-pCIneo.

## 3 Results

### 3.1 Expression properties of 11 mammalian ZACs in tsA201 cells

The abilities of *hs*ZAC and the 10 other mammalian ZACs to be expressed at the protein level as well as at the plasma membrane level in tsA201 cells were investigated in an ELISA. In all these HA-tagged subunits, the epitope was introduced extracellularly immediately before the first residue in the mature ZAC protein, since epitope-tagging of *hs*ZAC and other CLR subunits in this position has been found to be functionally silent (Krzywkowski et al., 2007; Jensen et al., 2013; Bracamontes et al., 2014; Gu et al., 2016; Madjroh et al., 2021a). In the ELISA, total expression levels (i.e., protein expressed at the cell surface and inside the cell) and cell surface expression levels of the ZAC proteins were determined in permeabilized (Triton-X-treated) and non-permeabilized tsA201 cells, respectively. In parallel control experiments, expression of HA-tagged 5-HT3B in tsA201 cells was found not to result in significant cell surface expression, whereas co-expression of HA-5-HT3B and 5-HT3A resulted in robust cell surface expression of the HA-5-HT3B (data not shown), demonstrating the ability of the ELISA to distinguish between cell surface and total expression of HA-tagged proteins (Davies et al., 1999; Boyd et al., 2003).

**FIGURE 2:**
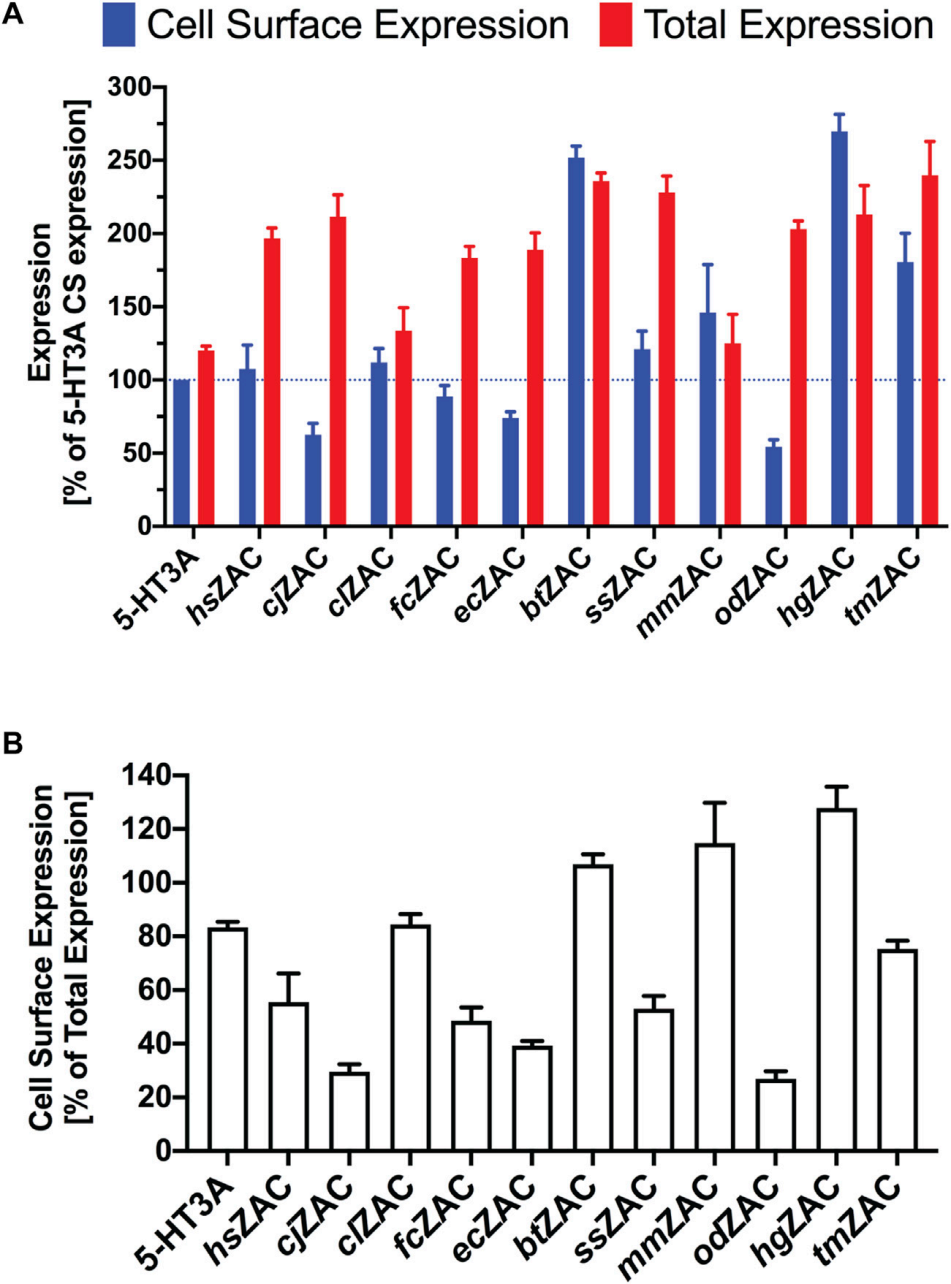
Expression properties displayed by 11 HA-tagged ZACs in tsA201 cells in an ELISA. **(A)**. Cell surface expression and total expression levels of *hs*ZAC, *cj*ZAC, *cl*ZAC, *fc*ZAC, *ec*ZAC, *bt*ZAC, *ss*ZAC, *mm*ZAC, HA-*od*ZAC, *hg*ZAC, and *tm*ZAC. Data are given as mean ± S.E.M. values in % of the cell surface expression in HA-tagged 5-HT3A-transfected cells (a line marker indicates the 100% level). **(B)**. Ratios between cell surface expression and total expression levels (CS-Exp/Total-Exp ratios) for all 11 ZACs. Data are given as mean ± S.E.M. in % of the total expression level of the respective receptors. **(A, B)**. Data are based on a total of three experiments (*n* = 3) performed in triplicate.

The total and cell surface expression levels displayed by *hs*ZAC and the other 10 mammalian ZACs in the ELISA are given in Figure 2A, where the expression levels are normalized to the cell surface expression exhibited by HA-tagged 5-HT3A (“CS-Exp_,5-HT3A_”). All 11 ZACs exhibited robust expression in the tsA201 cells, with total expression levels of the receptors ranging from 125% (*mm*ZAC) to 240% (*tm*ZAC) of CS-Exp,_5-HT3A_ (Figure 2A). All 11 HA-ZACs were also detected in significant levels at the plasma membrane level, with cell surface expression levels ranging from 54% (*od*ZAC) to 270% (*hg*ZAC) of CS-Exp,_5-HT3A_ (Figure 2A). The ability of the different HA-ZAC proteins to be transported to and be expressed at the cell surface differed substantially, as the ratios between cell surface expression and total expression levels (CS-Exp/Total-Exp) for the 11 HA-ZACs ranged from 0.27 to 0.39 (*od*ZAC, *cj*ZAC, *ec*ZAC), over 0.49–0.84 (*fc*ZAC, *ss*ZAC, *hs*ZAC, *tm*ZAC, *cl*ZAC), to 1.07–1.25 (*bt*ZAC, *mm*ZAC, *hg*ZAC) (Figure 2B). As for latter 3 ZACs, the cell surface expression and total expression levels displayed by *bt*ZAC and *mm*ZAC did not differ significantly, whereas *hg*ZAC displayed significantly higher cell surface expression than total expression. A CS-Exp/Total-Exp ratio >1 obviously contrasts with the definitions of cell surface expression and total expression. These high ratios are thus likely the result of experimental variation, minor effects of the Triton-X treatment on the cell populations for “total expression”, and/or a fraction of immature, unfolded or compartmentalized intracellular HA-tagged ZAC protein not being accessible for HA-antibody binding (thus leading to underestimation of the total expression level of the protein). Regardless of this, it is still reasonable to conclude that very high fractions of the *bt*ZAC, *mm*ZAC and *hg*ZAC proteins expressed in the tsA201 cells are trafficked to the cell surface level. In conclusion, transient transfection of cDNAs for these 11 mammalian ZACs in tsA201 cells resulted in robust protein expression and in significant cell surface expression of all the proteins.

### 3.2 Functional properties displayed by 7 mammalian ZACs in *Xenopus* oocytes in TEVC recordings

The significant cell surface expression exhibited by the 11 mammalian ZACs in the tsA201 cells strongly suggest that all of these subunits assemble into homomeric complexes in the plasma membrane. To address whether these ZAC assemblies are functional, the functional properties exhibited by Zn^2+^ and H^+^ at *hs*ZAC and six other mammalian ZACs were studied in *Xenopus* oocytes by TEVC recordings.

**FIGURE 3:**
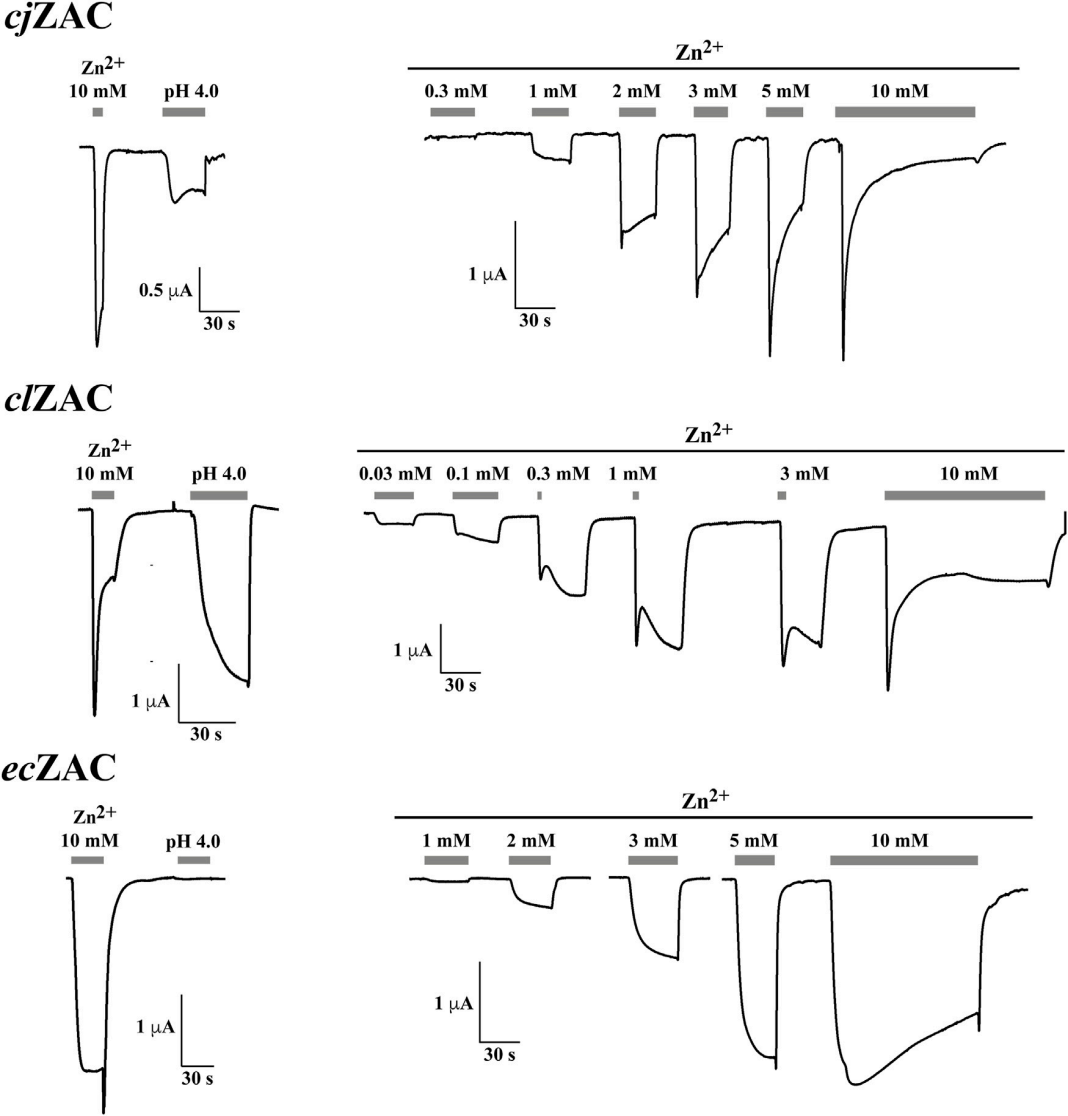
Functional properties displayed by *cj*ZAC (*top panel*), *cl*ZAC (*middle panel*) and *ec*ZAC (*bottom panel*) in *Xenopus* oocytes in two-electrode voltage-clamp recordings. *Left:* Representative traces for the current responses evoked by Zn^2+^ (10 mM) and H^+^ (pH 4.0) in oocytes expressing the receptors. *Right:* Representative traces for the current responses evoked by various Zn^2+^ concentrations in oocytes expressing the receptors. The averaged concentration-response relationships for the receptors are given in Figure 5, and averaged pharmacological data extracted from these experiments are given in Table 2.

**FIGURE 4:**
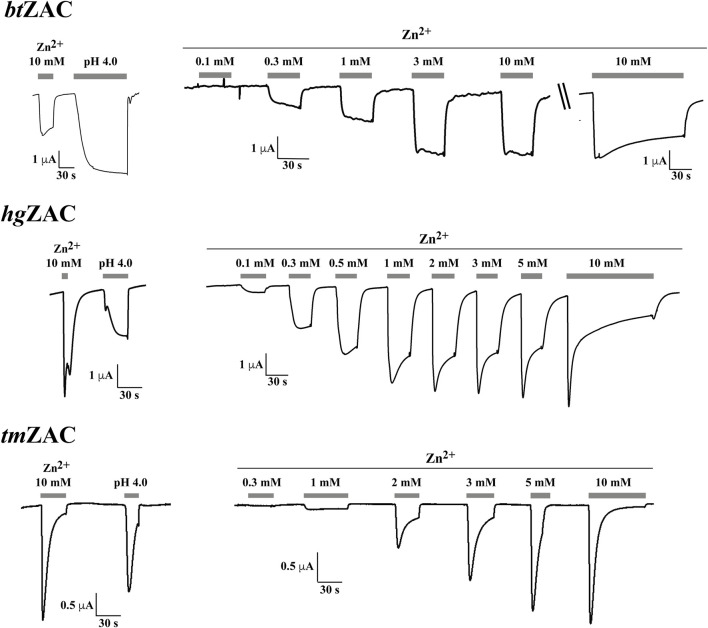
Functional properties displayed by *bt*ZAC (*top panel*), *hg*ZAC (*middle panel*) and *tm*ZAC (*bottom panel*) in *Xenopus* oocytes in two-electrode voltage-clamp recordings. *Left:* Representative traces for the current responses evoked by Zn^2+^ (10 mM) and H^+^ (pH 4.0) in oocytes expressing the receptors. *Right:* Representative traces for the current responses evoked by various Zn^2+^ concentrations in oocytes expressing the receptors. For *bt*ZAC, the last current response in the Zn^2+^ concentration-response trace is from another *bt*ZAC-expressing oocyte than that for the other current responses (indicated by “\\“). The averaged concentration-response relationships for the receptors are given in Figure 5, and averaged pharmacological data extracted from these experiments are given in Table 2.

**FIGURE 5:**
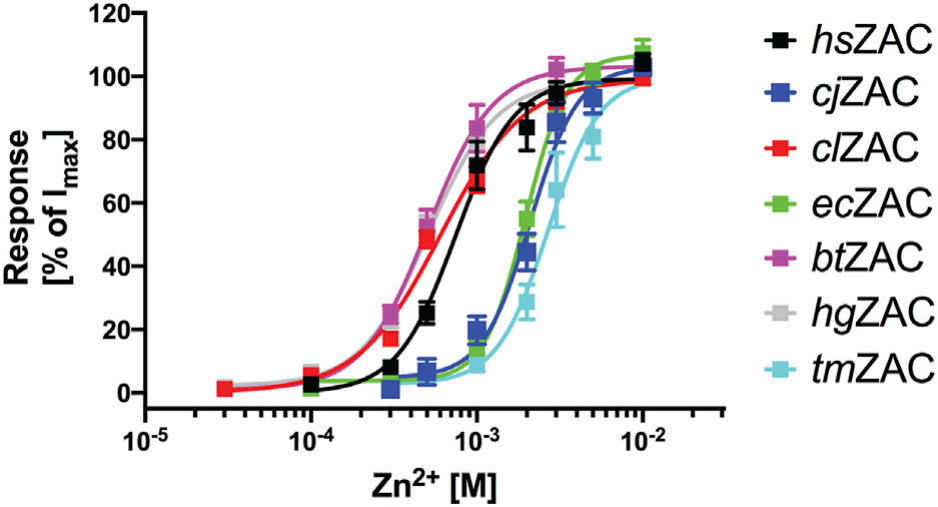
Concentration-response relationships exhibited by Zn^2+^ at *hs*ZAC, *cj*ZAC, *cl*ZAC, *ec*ZAC, *bt*ZAC, *hg*ZAC and *tm*ZAC in *Xenopus* oocytes in two-electrode voltage-clamp recordings. Data are given as mean ± S.E.M. values in % normalized to the fitted I_max_ for the specific receptor and are based on five to eight independent experiments (the specific n values for the receptors are given in Table 2). The averaged pharmacological data extracted from these experiments are given in Table 2.

Zn^2+^ (10 mM) mediated robust currents in oocytes expressing these 7 ZACs. Representative examples of current traces evoked by different Zn^2+^ concentrations are given in Figures 3, 4, the mean concentration-response relationships determined for the metal ion at the seven receptors are given in Figure 5, and the mean pharmacological properties extracted from the experiments are presented in Table 2. In agreement with previous studies of wild-type (untagged) human ZAC in oocytes (Madjroh et al., 2021a; Madjroh et al., 2021b; Madjroh et al., 2021c), Zn^2+^ displayed an EC_50_ value of 0.71 mM and a Hill slope of 2.6 ± 0.2 at *hs*ZAC (Table 2). Analogously, Zn^2+^ induced robust current responses in a concentration-dependent manner in oocytes expressing the six other mammalian ZACs and displayed comparable concentration-response relationships at these receptors (Figures 3–5). The EC_50_ values for Zn^2+^ at these 6 ZACs ranged from 0.46 mM (*bt*ZAC) to 2.8 mM (*tm*ZAC), and the Hill slopes of the fitted concentration-response curves also varied somewhat (from 1.9 to 3.6) (Figure 5; Table 2). With regard to the latter parameter, it should be noted that the concentration-response relationships characterized by the highest Hill slopes were observed for *cj*ZAC, *ec*ZAC and *tm*ZAC that also exhibited the highest Zn^2+^ EC_50_ values (Figure 5; Table 2). Thus, these higher nH values could at least in part be ascribed to curve fitting for these three receptors being based on concentration-response data characterized by less well-established saturation levels than for the other receptors (as it only was possible to test Zn^2+^ in concentrations up to 10 mM). The maximal current amplitudes evoked by a saturating Zn^2+^ concentration (I_10 mM Zn2+_) at the ZACs, determined at an even larger number of oocytes for each receptor than the Zn^2+^ concentration-response relationships, varied considerably. However, it should be noted that these were determined at oocytes injected with different cRNA quantities and incubated for either 2 or 3 days, and thus these values cannot be used for direct comparisons of the functional expression levels of the different receptors.

**TABLE 2:** Functional properties exhibited by 7 mammalian ZACs in *Xenopus* ooyctes determined by TEVC electrophysiology. EC_50_ values for Zn^2+^ are given in mM with pEC_50_ ± S.E.M. values in brackets, and the current amplitudes I_10 mM Zn2+_ and I_pH 4.0_ are given in μA. The numbers of experiments (n) underlying the data are given in superscript behind each value.

Receptor	Zn^2+^ EC_50_ (mM) [pEC_50_ ± S.E.M.] ^(n)^	Zn^2+^ n_H_ ± S.E.M. ^(n)^	I_10 mM Zn2+_ (μA) ^(n)^	I_pH 4.0_ (μA) ^(n)^
*hs*ZAC	0.71 [3.15 **±** 0.06] ^(8)^	2.5 **±** 0.2 ^(8)^	1.8 ± 0.3 ^(14)^	2.9 ± 0.5 ^(8)^
*cj*ZAC	2.0 [2.70 **±** 0.04] ^(6)^	3.2 **±** 0.5 ^(6)^	2.2 ± 0.5 ^(16)^	0.9 ± 0.3 ^(11)^
*cl*ZAC	0.54 [3.27 **±** 0.09] ^(8)^	1.9 **±** 0.2 ^(8)^	3.2 ± 0.7 ^(21)^	1.7 ± 0.5 ^(13)^
*ec*ZAC	2.1 [2.68 **±** 0.07] ^(7)^	3.6 **±** 0.2 ^(7)^	2.6 ± 0.4 ^(18)^	n.r. ^(8)^
*bt*ZAC	0.46 [3.34 **±** 0.12] ^(5)^	2.3 **±** 0.3 ^(5)^	2.4 ± 0.3 ^(18)^	4.9 ± 0.6 ^(6)^
*hg*ZAC	0.53 [3.27 **±** 0.05] ^(7)^	2.1 **±** 0.3 ^(7)^	5.0 ± 0.8 ^(15)^	1.3 ± 0.6 ^(8)^
*tm*ZAC	2.8 [2.59 **±** 0.06] ^(6)^	2.9 **±** 0.3 ^(6)^	2.0 ± 0.1 ^(11)^	0.8 ± 0.2 ^(5)^

n.r., no response: no significant current was observed upon pH 4.0 applications on these oocytes.

The ability of H^+^ to evoke currents in oocytes expressing the seven mammalian ZACs was also investigated. H^+^ (pH 4.0) elicited significant currents in oocytes expressing *hs*ZAC and five of the six other mammalian ZACs: *cj*ZAC, *cl*ZAC, *bt*ZAC, *hg*ZAC and *tm*ZAC (Figures 3, 4; Table 2). The I_pH 4.0_ values determined at the receptors varied considerably, but it should be stressed that they are not I_max_ values, since pH 4.0 is not a saturating concentration of H^+^ as ZAC agonist. Interestingly, in contrast to the robust H^+^-induced currents through the 6 ZACs, no significant currents were detected in *ec*ZAC-expressing oocytes upon application of pH 4.0 (Figure 3; Table 2).

Although TEVC recordings from oocytes do not allow for extraction of detailed information about the kinetic properties of ion channels, some interesting differences were observed between the current responses evoked by Zn^2+^ through the different ZACs (Figures 3, 4). The slow activation phase and the lack of significant decay that characterized the currents evoked by sub-maximal Zn^2+^ concentrations through both *ec*ZAC and *bt*ZAC was very similar to the current profile observed for *hs*ZAC [(Madjroh et al., 2021a) and data not shown]. In contrast, the currents evoked by sub-maximal Zn^2+^ concentrations at the four other ZACs were characterized by peak current being reached and by the onset of a visible decay phase within a ∼30 s-application (exemplified in Figures 3, 4). These differences were also reflected in the current decay profiles displayed by the six receptors in the presence of a saturating Zn^2+^ concentration. *cj*ZAC and in particular *tm*ZAC consistently displayed relatively fast decay, which resulted in very small levels of residual current after 2-min exposure to Zn^2+^ (10 mM). In contrast, the decay of the *ec*ZAC and *bt*ZAC currents were strikingly slower and characterized by low degrees of desensitization after the 2-min agonist application, with the current decay profiles exhibited by *cl*ZAC and *hg*ZAC being intermediate to these two sets of receptors in this respect (exemplified in Figures 3, 4).

## 4 Discussion

In the present study, ZACs from 10 different mammalian *ZACN* genomes other than human were demonstrated to be expressed at protein level and to traffic to the plasma membrane and assemble into cell surface-expressed complexes in mammalian cells. Moreover, six of these ZACs were found to form functional channels gated by Zn^2+^ and H^+^ in *Xenopus* oocytes. The specific differences in the expression and functional properties exhibited by the mammalian ZACs will be discussed further below, but overall, these findings suggest that ZAC functionality at the molecular level may be widely conserved throughout mammals.

The *ZACN* genes encoding for the 10 ZACs originate from a wide range of mammalian species, with ZACs from primates, carnivores, ungulates, bats, rodents and sirenians being represented (Table 1). Although this selection does not include representatives from all orders and families in the *Mammalia* class, it is nevertheless so diverse that it suggests that a *ZACN* gene encoding for a functional ZAC protein is widely conserved across mammals. The *Mammalia* class is characterized by an immense diversity, as its members through evolution have developed a wide range of physiological adaptations to exhibit a span of lifestyles (e.g., fossorial, aquatic, terrestrial, and arboreal), and the conservation of a functional ZAC across mammalian species may thus be indicative of a generalized physiological role for the receptor in the mammals. With specific regard to the notable absence of *ZACN* orthologs in the mouse and rat genomes, it is interesting to note that the coding regions of *ZACN* orthologs from at least some *Rodentia* members (naked mole-rat and common degus) produce ZAC proteins capable of assembling into cell-surface expressed functional receptors in heterologous expression systems. From a practical perspective, the demonstration of the functional ZAC protein encoded for by the *ZACN* ortholog in the naked mole-rat genome may suggest that this could be an attractive rodent model system for *in vivo* studies of ZAC functions, and the functional ZACs produced by the *ZACN* orthologs in the dog and pig genomes means that studies of the receptor in higher animals also may be possible.

The robust total and cell surface expression levels exhibited by all 11 mammalian ZACs expressed in tsA201 cells in the ELISA is a strong indication of evolutionary conservation of the *ZACN* orthologs in these species (Figure 2). In the ELISA, the 11 ZACs displayed substantially different degrees of cell surface expression (CS-Exp/Total-Exp ratios), and notably ZACs displaying comparable total expression levels in the tsA201 cells also displayed differential abilities to be transported to and assemble into ZAC complexes in the plasma membrane (see, for example, *cj*ZAC and *od*ZAC vs. *bt*ZAC and *hg*ZAC, Figure 2). These differences are clearly based in the molecular differences between the ZAC proteins and how these differences influence the intracellular processes underlying the folding and transport of the ZAC protein to the tsA201 cell plasma membrane. As interesting as these expression differences between the 11 ZACs in the isolated setting of this heterologous expression system may be, it is not possible to infer any solid conclusions about possible differences in the *in vivo* expression of the receptors in the respective mammalian species based on these data. However, the fact that the coding sequences of all 11 *ZACN* orthologs studied here produce robust expression of ZAC proteins in a mammalian cell line and that these proteins also traffic to and assemble into homomeric complexes in the plasma membrane of this cell strongly suggest that the functionality of ZAC at the molecular level is conserved in these mammals.

The functional properties exhibited by *cj*ZAC, *cl*ZAC, *ec*ZAC, *bt*ZAC, *hg*ZAC and *tm*ZAC expressed in *Xenopus* oocytes in the TEVC recordings were broadly similar to those displayed by *hs*ZAC (Figures 3, 4; Table 2). The EC_50_ values exhibited by Zn^2+^ at *cl*ZAC, *bt*ZAC and *hg*ZAC were very similar to its agonist potency at *hs*ZAC, whereas the Zn^2+^ EC_50_ values determined at *cj*ZAC, *ec*ZAC and *tm*ZAC were three to five fold higher (Figure 5; Table 2). I will refrain from speculating on whether these differences in Zn^2+^ potency between the recombinant ZACs could translate into different functional properties of the native ZACs in their respective mammals and on the potential implications of this. As will be addressed further below, it is also an open question whether the principal *in vivo* function of ZAC is as a zinc-sensor or whether the channel in fact mediates the signaling of another yet unidentified transmitter.

In concordance with the previously reported H^+^-mediated gating of human wild-type ZAC (Trattnig et al., 2016; Madjroh et al., 2021a) five of the other six other mammalian ZACs were found to be activated by protons (Figures 3, 4; Table 2). The contrasting inactivity of H^+^ as an agonist at *ec*ZAC at concentrations up to 100 μM (pH 4.0) is interesting, since the concentration-dependent activation of *ec*ZAC mediated by Zn^2+^ clearly demonstrate that this subunit forms functional channels in these oocytes (Figure 3). This lack of proton-evoked *ec*ZAC gating could be rooted in a lack of conservation of residues protonated in the other ZACs during their H^+^-evoked gating in this receptor. Three canonical amino acids, Glu, Asp and His (all have side chains with pK_a_ values of 4–7), are candidate residues for protonation at sub-physiological pH levels, and the vast majority of Glu, Asp and His residues in the *hs*ZAC protein are either completely conserved in the 7 ZACs (including *ec*ZAC) or are non-conserved in more than just *ec*ZAC of the 7 ZACs. Interestingly, however, there are four of these candidate residues that are completely conserved in the six H^+^-gated ZACs (*hs*ZAC, *cj*ZAC, *cl*ZAC, *bt*ZAC, *hg*ZAC and *tm*ZAC) but differ in the *ec*ZAC. More specifically, the Glu^136^, Glu^162^, Glu^235^ and Asp^397^ residues in the mature *hs*ZAC protein that correspond to Asp, Lys, Asp and Ala residues, respectively, in *ec*ZAC (Figure 1). In a previous study the extracellular domain of *hs*ZAC has been shown to contain key molecular determinants for the H^+^-evoked activation, but the importance of Glu^136^ and Glu^162^ in this domain for proton-mediated gating was not investigated (Madjroh et al., 2021b). The putative involvement of Glu^235^ in the transmembrane M2 α-helix and Asp^397^ in the extracellular carboxy-terminal of *hs*ZAC have not been investigated either, which makes these four residues interesting candidates for future investigations into the molecular mechanisms underlying H^+^-evoked ZAC gating. However, the inability of *ec*ZAC to be activated by H^+^ does not necessarily arise from the absence of these non-conserved putative protonation residues in the subunit. The proton-evoked gating of a pLGIC from the bacteria *Gloeobacter violaceus*, GLIC, has been proposed to involve an initial protonation of a Glu in the extracellular domain of the receptor leading to formation of water-mediated hydrogen-bond networks through electrostatic triads of residues throughout the pentameric complex resulting in opening of the ion channel (Nemecz et al., 2017; Hu et al., 2018). If a similar complex process is underlying H^+^-induced ZAC activation, molecular differences (compared to the other ZACs) in other regions of *ec*ZAC than the protonated residue(s) could result in this initial protonation step not being translated into channel gating in this specific receptor.

Considering the markedly different current response profiles exhibited by the 7 ZACs in the TEVC recordings (Figures 3, 4), it would also be interesting to probe the importance of non-conserved residues between ZACs such as *tm*ZAC and *bt*ZAC that display very distinct current response profiles in future studies. Whether the different pharmacological and channel properties exhibited by the recombinant channels in the oocyte recordings translate into substantial differences in the signaling properties of native ZACs in their respective mammals is another question. While ZAC seems to be the only member of the fifth mammalian pLGIC subfamily, it cannot be completely ruled out that it could assembly into heteromeric complexes with other members of this superfamily, and complex formation of ZAC with other transmembrane or intracellular proteins or *in vivo* phosphorylation of residues in its intracellular domains could also be envisioned to give rise to significantly different functional properties of the native ZAC.

The observed modest differences in Zn^2+^ agonist potencies between the 7 ZACs, the non-responsiveness of *ec*ZAC to H^+^, and the different current responses evoked by Zn^2+^ and H^+^ through the receptors in the TEVC recordings also warrant a consideration about whether Zn^2+^, Cu^2+^ and/or H^+^ are in fact the principal endogenous ZAC agonist(s). Because of their markedly different sizes and physicochemical properties compared to GABA, glycine, serotonin, and ACh, the three identified ZAC agonists most likely do not target the sites in ZAC corresponding to the orthosteric sites in the classical pLGICs. Based on the substantial spontaneous activity exhibited by *hs*ZAC and the numerous allosteric sites for Zn^2+^, Cu^2+^ and H^+^ identified in other pLGICs (Lovinger, 1991; Bloomenthal et al., 1994; Laube et al., 1995; Palma et al., 1998; Harvey et al., 1999), it has been proposed that the metal ion- and proton-induced ZAC gating could be mediated through allosteric sites through a potentiation of this constitutive activity (Madjroh et al., 2021b). In this scenario, a possible lack of evolutionary pressure to conserve the compositions of this/these allosteric site(s) in ZAC could potentially be at the root of some of the differences in Zn^2+^ and H^+^ pharmacology and possibly also to the apparent kinetic differences between the 7 ZACs. Importantly, even if this study thus not necessarily has investigated ZAC functionality using its “true” orthosteric agonist, the activation of the 7 ZACs mediated by Zn^2+^ and H^+^ nevertheless demonstrates the functionality of the receptors.

In conclusion, the ability of a range of mammalian *ZACN* genes to produce proteins capable of assembling into functional receptor complexes in heterologous expression systems strongly suggests that ZAC functionality at the molecular level is widely conserved within mammals. This finding supports the notion that ZAC may govern specific physiological functions in mammals, including humans, and hopefully it will prompt further investigation into what these functions could be.

## Data Availability

The original contributions presented in the study are included in the article, further inquiries can be directed to the corresponding author.

## References

[B1] Alexander S. P. Mathie A. Peters J. A. Veale E. L. Striessnig J. Kelly E. (2021). The concise guide to pharmacology 2019/20: ion channels. Br. J. Pharmacol. 178 (1), S142–S228. 10.1111/bph.14749 PMC6844578 31710715

[B2] Almagro Armenteros J. J. Tsirigos K. D. Sønderby C. K. Petersen T. N. Winther O. Brunak S. (2019). SignalP 5.0 improves signal peptide predictions using deep neural networks. Nat. Biotechnol. 37, 420–423. 10.1038/s41587-019-0036-z 30778233

[B3] Belelli D. Hogenkamp D. Gee K. W. Lambert J. J. (2020). Realising the therapeutic potential of neuroactive steroid modulators of the GABA_A_ receptor. Neurobiol. Stress. 12, 100207. 10.1016/j.ynstr.2019.100207 32435660 PMC7231973

[B4] Bloomenthal A. B. Goldwater E. Pritchett D. B. Harrison N. L. (1994). Biphasic modulation of the strychnine-sensitive glycine receptor by Zn^2+^ . Mol. Pharmacol. 46, 1156–1159. 7808436

[B5] Boyd G. W. Doward A. I. Kirkness E. F. Millar N. S. Connolly C. N. (2003). Cell surface expression of 5-hydroxytryptamine type 3 receptors is controlled by an endoplasmic reticulum retention signal. J. Biol. Chem. 278 (30), 27681–27687. 10.1074/jbc.M304938200 12750374

[B6] Bracamontes J. R. Li P. Akk G. Steinbach J. H. (2014). Mutations in the main cytoplasmic loop of the GABA_A_ receptor α4 and δ subunits have opposite effects on surface expression. Mol. Pharmacol. 86 (1), 20–27. 10.1124/mol.114.092791 24723490 PMC4054001

[B7] Chang Y. (2019). Modulators of zinc Activated Channel (US 2019/0022121 A1). Phoeniz, AZ, United States: Dignity Health.

[B8] Chua H. C. Chebib M. (2017). GABA_A_ receptors and the diversity in their structure and pharmacology. Adv. Pharmacol. 79, 1–34. 10.1016/bs.apha.2017.03.003 28528665

[B9] Davies P. A. Pistis M. Hanna M. C. Peters J. A. Lambert J. J. Hales T. G. (1999). The 5-HT_3B_ subunit is a major determinant of serotonin-receptor function. Nature 397 (6717), 359–363. 10.1038/16941 9950429

[B10] Davies P. A. Wang W. Hales T. G. Kirkness E. F. (2003). A novel class of ligand-gated ion channel is activated by Zn^2+^ . J. Biol. Chem. 278 (2), 712–717. 10.1074/jbc.M208814200 12381728

[B11] Fakhfouri G. Rahimian R. Dyhrfjeld-Johnsen J. Zirak M. R. Beaulieu J. M. (2019). 5-HT_3_ receptor antagonists in neurologic and neuropsychiatric disorders: the iceberg still lies beneath the surface. Pharmacol. Rev. 71 (3), 383–412. 10.1124/pr.118.015487 31243157

[B12] Ghasemi M. Hadipour-Niktarash A. (2015). Pathologic role of neuronal nicotinic acetylcholine receptors in epileptic disorders: implication for pharmacological interventions. Rev. Neurosci. 26 (2), 199–223. 10.1515/revneuro-2014-0044 25565544

[B13] Gibbs E. Chakrapani S. (2021). Structure, function and physiology of 5-hydroxytryptamine receptors subtype 3. Subcell. Biochem. 96, 373–408. 10.1007/978-3-030-58971-4_11 33252737

[B14] Gu S. Matta J. A. Lord B. Harrington A. W. Sutton S. W. Davini W. B. (2016). Brain α7 nicotinic acetylcholine receptor assembly requires NACHO. Neuron 89 (5), 948–955. 10.1016/j.neuron.2016.01.018 26875622

[B15] Harvey R. J. Thomas P. James C. H. Wilderspin A. Smart T. G. (1999). Identification of an inhibitory Zn^2+^ binding site on the human glycine receptor α1 subunit. J. Physiol. 520, 53–64. 10.1111/j.1469-7793.1999.00053.x 10517800 PMC2269571

[B16] Houtani T. Munemoto Y. Kase M. Sakuma S. Tsutsumi T. Sugimoto T. (2005). Cloning and expression of ligand-gated ion-channel receptor L2 in central nervous system. Biochem. Biophys. Res. Commun. 335 (2), 277–285. 10.1016/j.bbrc.2005.07.079 16083862

[B17] Hu H. Ataka K. Menny A. Fourati Z. Sauguet L. Corringer P. J. (2018). Electrostatics, proton sensor, and networks governing the gating transition in GLIC, a proton-gated pentameric ion channel. Proc. Natl. Acad. Sci. U.S.A. 115 (52), E12172–E12181. 10.1073/pnas.1813378116 30541892 PMC6310827

[B18] Jensen A. B. Hoestgaard-Jensen K. Jensen A. A. (2013). Elucidation of molecular impediments in the α6 subunit for *in vitro* expression of functional α6β4* nicotinic acetylcholine receptors. J. Biol. Chem. 288, 33708–33721. 10.1074/jbc.M113.509356 24085295 PMC3837116

[B19] Juza R. Vlcek P. Mezeiova E. Musilek K. Soukup O. Korabecny J. (2020). Recent advances with 5-HT_3_ modulators for neuropsychiatric and gastrointestinal disorders. Med. Res. Rev. 40, 1593–1678. 10.1002/med.21666 32115745

[B20] Krzywkowski K. Davies P. A. Feinberg-Zadek P. L. Bräuner-Osborne H. Jensen A. A. (2008). A high frequency *HTR3B* variant associated with major depression dramatically augments the signaling of the human 5-HT_3AB_ receptor. Proc. Natl. Acad. Sci. U.S.A. 105, 722–727. 10.1073/pnas.0708454105 18184810 PMC2206603

[B21] Krzywkowski K. Jensen A. A. Connolly C. N. Bräuner-Osborne H. (2007). Naturally occurring mutations in the human 5-HT_3A_ gene profoundly impact 5-HT_3_ receptor function and expression. Pharmacogenet. Genomics 17, 255–266. 10.1097/FPC.0b013e3280117269 17496724

[B22] Laube B. Kuhse J. Rundstrom N. Kirsch J. Schmieden V. Betz H. (1995). Modulation by zinc ions of native rat and recombinant human inhibitory glycine receptors. J. Physiol. 83, 613–619. 10.1113/jphysiol.1995.sp020610 PMC1157806 7776247

[B23] Lovinger D. M. (1991). Inhibition of 5-HT_3_ receptor-mediated ion current by divalent metal cations in NCB-20 neuroblastoma cells. J. Neurophysiol. 66, 1329–1337. 10.1152/jn.1991.66.4.1329 1722246

[B24] Lynch J. W. Zhang Y. Talwar S. Estrada-Mondragon A. (2017). Glycine receptor drug discovery. Adv. Pharmacol. 79, 225–253. 10.1016/bs.apha.2017.01.003 28528670

[B25] Madjroh N. Davies P. A. Smalley J. L. Kristiansen U. Söderhielm P. C. Jensen A. A. (2021a). Delineation of the functional properties exhibited by the Zinc-Activated Channel (ZAC) and its high-frequency Thr128Ala variant (rs2257020) in *Xenopus* oocytes. Pharmacol. Res. 169, 105653. 10.1016/j.phrs.2021.105653 33962015 PMC8248299

[B26] Madjroh N. Mellou E. Æbelø L. Davies P. A. Söderhielm P. C. Jensen A. A. (2021b). Probing the molecular basis for signal transduction through the Zinc-Activated Channel (ZAC). Biochem. Pharmacol. 193, 114781. 10.1016/j.bcp.2021.114781 34560053 PMC11359809

[B27] Madjroh N. Mellou E. Davies P. A. Söderhielm P. C. Jensen A. A. (2021c). Discovery and functional characterization of N-(thiazol-2-yl)-benzamide analogs as the first class of selective antagonists of the Zinc-Activated Channel (ZAC). Biochem. Pharmacol. 193, 114782. 10.1016/j.bcp.2021.114782 34560054 PMC9979163

[B28] Nemecz A. Hu H. Fourati Z. Van Renterghem C. Delarue M. Corringer P. J. (2017). Full mutational mapping of titratable residues helps to identify proton-sensors involved in the control of channel gating in the *Gloeobacter violaceus* pentameric ligand-gated ion channel. PLoS Biol. 15 (12), e2004470. 10.1371/journal.pbio.2004470 29281623 PMC5760087

[B29] Palma E. Maggi L. Miledi R. Eusebi F. (1998). Effects of Zn^2+^ on wild type and mutant neuronal α7 nicotinic receptors. Proc. Natl. Acad. Sci. U.S.A. 95, 10246–10250. 10.1073/pnas.95.17.10246 9707632 PMC21493

[B30] Taly A. Corringer P. J. Guedin D. Lestage P. Changeux J. P. (2009). Nicotinic receptors: allosteric transitions and therapeutic targets in the nervous system. Nat. Rev. Drug Discov. 8 (9), 733–750. 10.1038/nrd2927 19721446

[B31] Terry A. V. Jr. Callahan P. M. (2020). α7 nicotinic acetylcholine receptors as therapeutic targets in schizophrenia: update on animal and clinical studies and strategies for the future. Neuropharmacology 170, 108053. 10.1016/j.neuropharm.2020.108053 32188568 PMC8141324

[B32] Trattnig S. M. Gasiorek A. Deeb T. Z. Ortiz E. J. Moss S. J. Jensen A. A. (2016). Copper and protons directly activate the zinc-activated channel. Biochem. Pharmacol. 103, 109–117. 10.1016/j.bcp.2016.02.004 26872532 PMC5119521

